# Modern system approach to the diagnosis, therapy and rehabilitation of mental disorders

**DOI:** 10.1192/j.eurpsy.2021.1360

**Published:** 2021-08-13

**Authors:** V. Mitikhin, T. Solokhina, G. Tiumenkova

**Affiliations:** Department Of Mental Health Support Systems Research Centre, Mental Health Research Centre, Moscow, Russian Federation

**Keywords:** mental, disorders, system analysis, analytic hierarchy process

## Abstract

**Introduction:**

The current stage of research on mental disorders is associated with the use of system approaches to the development of the scientific foundations of psychiatric care.

**Objectives:**

Approach to solving problems that arise in the diagnosis of psychopathological conditions, assessing their severity, as well as evaluating the effectiveness of psychosocial treatment and rehabilitation.

**Methods:**

Clinical, psychometric, system analysis methods and algorithms of the Analytical Hierarchy Process (AHP) [1] were used.

**Results:**

When assessing a patient’s condition and behavior, it is necessary to make decisions (diagnosis, development of treatment and rehabilitation plans) based on heterogeneous information (genetic, neuronal and environmental, involving individual characteristics, as well as family and social context). This information is hierarchically organized and includes quantitative and qualitative data. Exposure at each of these different levels can affect the onset and course of the disease, and therefore should be considered in primary prevention and subsequent psychosocial therapy and rehabilitation of patients. Analysis of the problems of assessing psychopathological states and related psychosocial problems shows that these problems can be presented in the form of appropriate hierarchies, the structure of which must be taken into account when processing the initial information. The main advantages of the AHP include the use of the relationship scale (fundamental scale) for processing heterogeneous data based on expert, clinical information.

**Conclusions:**

The approach provides correct integration of heterogeneous characteristics when considering diagnostic procedures, psychosocial therapy and rehabilitation.1. Mitikhin V.G., Solokhina T.A. S.S. Korsakov Journal of Neurology and Psychiatry, 2019, 2: 49-54. doi:10.17116/jnevro201911902149

